# Effects of Chronic Heat Stress on Growth, Apoptosis, Antioxidant Enzymes, Transcriptomic Profiles, and Immune-Related Genes of Hong Kong Catfish (*Clarias fuscus*)

**DOI:** 10.3390/ani14071006

**Published:** 2024-03-26

**Authors:** Yong Liu, Changxu Tian, Zhihua Yang, Cailin Huang, Kaizhi Jiao, Lei Yang, Cunyu Duan, Zhixin Zhang, Guangli Li

**Affiliations:** 1 Guangdong Research Center on Reproductive Control and Breeding Technology of Indigenous Valuable Fish Species, Guangdong Provincial Engineering Laboratory for Mariculture Organism Breeding, Guangdong Provincial Key Laboratory of Aquatic Animal Disease Control and Healthy Culture, Fisheries College, Guangdong Ocean University, Zhanjiang 524088, China; 2112101115@stu.gdou.edu.cn (Y.L.); tiancx@gdou.edu.cn (C.T.); yzh2798139505@163.com (Z.Y.); 2112101009@stu.gdou.edu.cn (K.J.); yl172022@163.com (L.Y.); duancherry77@163.com (C.D.); 2 Guangxi Introduction and Breeding Center of Aquaculture, Nanning 530001, China; hcl201809@163.com (C.H.); 18934984755@163.com (Z.Z.)

**Keywords:** transcriptome, oxidative stress, chronic heat stress, apoptosis, *Clarias fuscus*

## Abstract

**Simple Summary:**

The Hong Kong catfish (*Clarias fuscus*) is a tropical and subtropical fish that is extensively cultured in southern China due to its adaptability and tolerance. While previous studies have investigated the impact of global-warming-induced increased water temperature on teleost fish, the response of *C. fuscus* to chronic high temperature and its underlying molecular mechanism remains unclear. This study conducted a 90-day heat-stress experiment on *C. fuscus*, revealing that continuous exposure to high temperatures stunted their growth, caused liver tissue damage, and increased apoptosis rates. Additionally, changes in antioxidant enzyme activity and an upregulation of heat shock protein (HSP) genes were observed, indicating a potential immune response mechanism. These results suggest that *C. fuscus* may counteract the negative effects of prolonged high-temperature stress by upregulating heat-shock-protein-related genes. This research enhances our comprehension of *C. fuscus*’ molecular responses to high-temperature stress and serves as a valuable model for studying similar responses in other fish species.

**Abstract:**

Chronic heat stress can have detrimental effects on the survival of fish. This study aimed to investigate the impact of prolonged high temperatures on the growth, antioxidant capacity, apoptosis, and transcriptome analysis of Hong Kong catfish (*Clarias fuscus*). By analyzing the morphological statistics of *C. fuscus* subjected to chronic high-temperature stress for 30, 60, and 90 days, it was observed that the growth of *C. fuscus* was inhibited compared to the control group. The experimental group showed a significant decrease in body weight and body length compared to the control group after 60 and 90 days of high-temperature stress (*p* < 0.05, *p* < 0.01). A biochemical analysis revealed significant alterations in the activities of three antioxidant enzymes superoxide dismutase activity (SOD); catalase activity (CAT); glutathione peroxidase activity (GPx), the malondialdehyde content (MDA), and the concentrations of serum alkaline phosphatase (ALP); Aspartate aminotransferase (AST); and alanine transaminase (ALT) in the liver. TUNEL staining indicated stronger apoptotic signals in the high-temperature-stress group compared to the control group, suggesting that chronic high-temperature-induced oxidative stress, leading to liver tissue injury and apoptosis. Transcriptome analysis identified a total of 1330 DEGs, with 835 genes being upregulated and 495 genes being downregulated compared to the control group. These genes may be associated with oxidative stress, apoptosis, and immune response. The findings elucidate the growth changes in *C. fuscus* under chronic high temperature and provide insights into the underlying response mechanisms to a high-temperature environment.

## 1. Introduction

Changes in environmental temperatures have significant effects on fish survival and physiological functions. In recent years, the aquaculture industry has been facing unprecedented challenges due to the rise in global average temperatures and an increase in extreme heatwave events driven by climate change [1]. Models by researchers such as Braz-Mota et al. predict that the global average surface temperature could increase by up to 6 °C by the end of this century [2]. While fish can adapt within a certain temperature range, excessive environmental temperatures can lead to a variety of adverse physiological changes, including pathological tissue cell hyperplasia and complex metabolic stress responses [3,4,5,6]. In severe cases, these changes can result in mortality [7]. These temperature-induced changes threaten not only the health of individual fish but also the viability of fish populations and the economic sustainability of the aquaculture industry. Studies on the physiological responses to temperature fluctuations in species such as *Cyprinus carpio* [8], *Oncorhynchus mykiss* [9], and *Oryzias latipes* [10] have illustrated the potential impacts on aquatic ecosystems. Consequently, it is critically important to investigate the mechanisms underlying thermotolerance in fish [11].

Under high-temperature exposure, fish experience an increase in the production of anaerobic free radicals, which can lead to oxidative stress and subsequent toxic effects due to the accumulation of oxidative byproducts [12,13]. To counteract the excessive formation of reactive oxygen species (ROS) under heat stress, aquatic animals can activate their antioxidant defense mechanisms. This system relies on small reductive molecules such as glutathione and ascorbic acid, alongside enzymes like superoxide dismutase (SOD), catalase (CAT), and glutathione peroxidase (GPx) [14,15,16]. These enzymes are crucial for cellular protection against ROS, with SOD converting oxygen to hydrogen peroxide, CAT breaking down hydrogen peroxide into water and oxygen, and GPx addressing lipid peroxidation byproducts [17]. The complex interplay of enzymatic and non-enzymatic antioxidants forms a network that serves as a first line of defense, particularly SOD, against oxidative stress [18,19,20]. Enhanced activities of these antioxidant enzymes are typically indicative of an organism's adaptive response to oxidative challenges, effectively reducing ROS levels [21]. Nevertheless, there are instances where this antioxidant response is insufficient to prevent oxidative damage, as evidenced in the liver of pikeperch (*Sander lucioperca*) under acute thermal stress [22]. Currently, the physiological responses of Hong Kong catfish to heat stress and their capacity to mitigate oxidative stress remain unexplored.

The liver, as a principal immunometabolic organ in fish, plays a critical role in the regulation of metabolism and the orchestration of numerous physiological processes, particularly under elevated temperature conditions [23]. When subjected to thermal stress, alterations in the cellular architecture of the fish liver occur, impeding its capacity for substance synthesis and transport. This disturbance leads to significant metabolic imbalances, manifesting as altered rates of oxygen consumption, ammonia excretion, and phosphorus excretion [24]. Numerous studies have demonstrated that environmental stresses can affect liver structure and function [22]. For example, exposure to high temperatures can induce oxidative stress in the liver of the red cusk-eel (*Genypterus chilensis*), causing oxidative damage and triggering transcriptional regulation of the antioxidant response [25]. Additionally, heat stress has been found to cause liver injury in white pike-kissing perch, characterized by vacuolar degeneration and nucleolysis [22]. Zhao et al. [26] have also shown that high-temperature conditions induce liver injury and apoptosis in largemouth bass. Nevertheless, the impact of sustained thermal stress on the structural integrity and physiological function of the liver in Hong Kong catfish remains unreported.

Temperature stress primarily induces a series of physiological and biochemical changes through the modulation of gene expression [27]. Understanding the alterations in gene expression is crucial for unraveling the mechanisms underlying adaptation to high temperatures. Transcriptomics, as a powerful tool for identifying gene changes, facilitates the exploration of temperature stress-induced gene expression alterations. Numerous studies have employed transcriptomic approaches to investigate the patterns of gene expression and their regulatory mechanisms in various fish species exposed to heat stress [26,28,29,30], such as Atlantic salmon (*Salmo salar*), rainbow trout (*Oncorhynchus mykiss*), half-smooth tongue sole (*Cynoglossus semilaevis*), and snow trout (*Schizothorax richardsonii*). These findings provide valuable insights into the molecular basis of fish adaptation to high-temperature stress.

The family Clariidae, which belongs to the class Actinopteri and order Siluriformes, is widely distributed in freshwater regions of Africa and Asia [31]. They inhabit a variety of natural habitats and are well-adapted to changes in temperature [32]. The Hong Kong catfish (*Clarias fuscus*) is the only native fish of the Clariidae family in China [31]. It is a tropical and subtropical fish, with a survival water temperature range of 10 to 32 °C and an optimum growth temperature range of 25 to 30 °C [33]. This species is an economically important bottom freshwater fish widely cultured in southern China, known for its strong adaptability and tolerance [34,35,36]. However, even the well-adapted and tolerant Siluriformes have been affected to some extent by the increasing water temperature caused by global climate change [33,37]. It is still unknown how different living environment temperatures will affect the response of Siluriformes to continuous high temperatures. The *C. fuscus* are usually able to survive and reproduce at higher water temperatures and are more adaptable to high temperatures. In this study, we took *C. fuscus* as the research object, aiming to investigate its adaptive ability and physiological mechanism under a high-temperature environment. It is necessary to select both fish species resistant to water temperature and sensitive species for the study to investigate the effects of a high-temperature environment and adaptive mechanisms on fish from different angles. Studying the survival and physiological conditions of sensitive species under high water temperatures can help us to better understand the mechanisms of biological tolerance and adaptation to high temperatures. In this study, we analyzed four aspects: morphology, antioxidant enzyme activity, apoptosis, and transcriptome response, to evaluate the effects of continuous high temperatures on the molecular mechanisms of *C. fuscus*. The results provide preliminary insights into the growth changes in *C. fuscus* under continuous high temperatures and the underlying response mechanisms to high-temperature environments.

## 2. Materials and Methods

### 2.1. Ethics Statement

All experimental protocols in this study were approved by the Animal Research and Ethics Committee of Guangdong Ocean University (NIH Pub. No. 85–23, revised 1996). This study does not involve endangered or protected species.

### 2.2. Experimental Design and Management

The *C. fuscus* fry (body weight 1 ± 0.2 g, 1 month old) were randomly selected and purchased from Guangxi Hongtai Aquatic Product Farm, Beiliu, Guangxi Province, China. After a week of temporary culture at the freshwater aquaculture base of Guangdong Ocean University, the fish were randomly divided into two groups: experimental group (34 ± 0.5 °C, HT) and control group (26 ± 2 °C, CT) (three parallels in each group). They were then cultured for 90 days. The initial water temperature of the experimental group was 28 °C, and it was gradually increased at a rate of 1 °C per hour until reaching 34 °C. Both groups were fed twice a day at fixed times. The feed is Fenghua brand *C. fuscus* feed No. 1 and No. 2. The feed type is floating particles. At 30, 60, and 90 days, 10 fish were randomly selected from each parallel of the two groups, anesthetized with MS-222 (Sigma, Livonia, MI, USA), and their body length and body weight were measured. Six serum and liver tissue samples were taken from each group of fish (*n* = 6). One portion of liver tissue was stored in a −80 °C refrigerator for oxidative stress analysis and RNA-seq and the other portion was stored in Bourne's reagent for TUNEL fluorescence section observation.

### 2.3. Morphological Statistics

The growth traits of *C. fuscus* were measured in both the high-temperature experimental group and control group on days 30, 60, and 90. The total body length was measured accurately using calipers with a precision of 0.01 mm, and the body weight was determined using an electronic scale with a precision of 0.01 mg after gently wiping off any surface moisture from the fish. The collected data were statistically analyzed using GraphPad software (8.0.2) to evaluate the effects of high temperatures on the growth and development of the *C. fuscus*.

### 2.4. TUNEL Staining of Liver Tissue for Apoptosis Detection

The liver tissues were dehydrated using graded alcohols, cleared in xylene, and infiltrated with wax. The tissues were then embedded in paraffin using an automatic tissue embedding machine (Leica, Wetzlar, Germany) and placed in a refrigerator at −20 °C for the paraffin wax to solidify. Subsequently, the paraffin blocks were sliced to a thickness of 5–8 μm using a rotary slicer RM2235 (Leica, Wetzlar, Germany) and transferred to slides for drying. For apoptosis detection, the Fluorescein (FITC) TUNEL Cell Apoptosis Detection Kit (Servicebio, Wuhan, China) was used, following the manufacturer’s protocol. The UV excitation wavelength (excitation spectrum, EX) of DAPI was 330–380 nm, emitting blue light (emission spectrum, EM: 420 nm). FITC had an EX of 465–495 nm, emitting red light (EM: 515–555 nm), and the nuclei of positive apoptotic cells appeared red. To preserve fluorescence, the sections were sealed with an anti-fade mounting medium. Fluorescence microscopy was employed to observe the stained tissues, and images were captured for further analysis.

### 2.5. Serum and Liver Indexes Test

The activities of alkaline phosphatase (ALP), aspartate aminotransferase (AST), alanine aminotransferase (ALT), catalase (CAT), superoxide dismutase (SOD), glutathione peroxidase (GPx), and malonaldehyde (MDA) contents were assessed using the manufacturer’s instructions. To minimize interference, three biological replicates and two technical replicates were performed on each serum and liver sample. The measurement steps were carried out according to the instructions provided with the kit.

### 2.6. RNA Extraction, Library Construction, Sequencing and Assembly

RNA extraction from liver tissues was performed using the TRIzol Reagent Kit (Thermo Fisher Scientific Inc., Waltham, MA, USA), following the manufacturer’s instructions. The degradation and contamination of RNA were determined by 1% agarose gel electrophoresis. The integrity and total amount of RNA were further detected by Agilent bioanalyzer 2100 system [38]. The NEBNext^®^ Ultra^TM^ RNA Library Prep Kit (NEB, Ipswich, MA, USA) was used to establish the RNA library according to the manufacturer's protocol. After library construction, the library was quantified using the Qubit2.0 Fluorometer (Thermo Fisher Scientific, Waltham, MA, USA) and diluted to a concentration of 1.5 ng/μL. Then the insert size of the library was detected by the Agilent 2100 bioanalyzer (Agilent, Beijing, China). Subsequently, the effective concentration of the library was accurately quantified by qRT-PCR (the effective concentration of the library was higher than 2 nM) to ensure the quality of the library. The sequencing was performed using the Illumina NovaSeqTM 6000 (Illumina, San Diego, CA, USA), generating 150 bp paired end reads. The original reads in the fastq format were filtered to remove the reads with adaptors, the reads with >10 % unknown nucleotides, and low-quality reads (>50% bases with a Q-value ≤ 20) and finally assembled by Trintey software (v2.15.0) using the default parameters to obtain clean reads. An index of the reference genome was constructed using HISAT2 v2.0.5 [39], and the paired-end clean reads were aligned to the reference genome [31]. The RNA-Seq data were deposited into the NCBI SRA database with the accession number PRJNA988888.

### 2.7. Screening of Differentially Expressed Genes (DEGs) and Functional Enrichment Analysis

The feature Counts tool software in subread (2.0.4) software was used to analyze the gene expression of each sample. The fragment per kilobase of transcript per million mapped reads (FPKM) was calculated based on the length of the gene to estimate the expression level. DESeq2 software [40] (1.20.0) was used to identify DEGs with the criteria for screening differentially expressed genes were |log_2_FC| ≥ 2 and *p* < 0.05 between each comparison group. The Cluster Profiler (3.8.1) software was used to perform Gene Ontology (GO) functional enrichment analysis and Kyoto Encyclopedia of Genes and Genomes (KEGG) pathway enrichment analysis on all DEGs.

### 2.8. Real-Time Fluorescence Quantitative PCR (qPCR) Validation

In this study, the accuracy of the transcriptome screening results was verified using qPCR. qPCR was performed on a real-time fluorescence quantitative PCR instrument (Roche, Basel, Switzerland), and the reaction system and conditions were as described in the Fluorescence Quantification Kit (TransGen, Beijing, China). Ten target gene expression levels were quantified using the 2^−ΔΔCt^ method with β-actin serving as the internal control. The statistical analyses, including ANOVA and Duncan's multiple range test, were performed using SPSS 24.0, with significance set at *p* < 0.05. The primer sequences were designed using Primer Premier 5 based on the sequencing results (Table 1).

### 2.9. Statistical Analysis

All the data were plotted using GraphPad Prism 9, which was used for drawing, and SPSS 24.0 software was used for significant difference analysis, including ANOVA and Duncan's multiple range test, with the significance set at *p* < 0.05.

## 3. Results

### 3.1. Morphological Statistics

**Figure 1:**
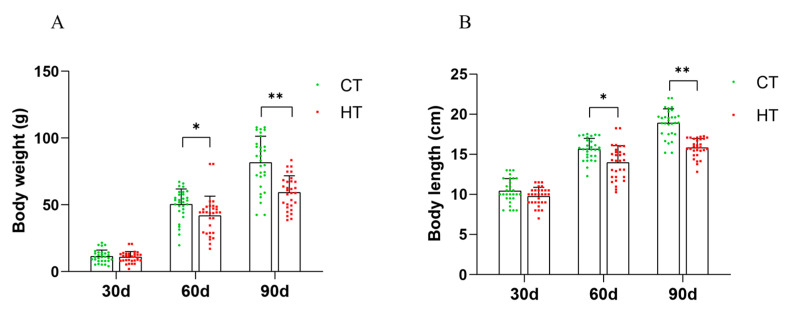
Growth indices of *C. fuscus* at different time points during chronic heat stress. (**A**) body weight; (**B**) HT: high-temperature group; CT: control group. *n* = 30; * indicates *p* < 0.05; ** indicates *p* < 0.01.

The results indicate that chronic heat stress had a significant inhibitory effect on the growth of *C. fuscus*. The experimental group showed a significant decrease in body weight and body length compared to the control group after 60 and 90 days of treatment (*p* < 0.05). However, the growth traits of the 30-day treatment group did not exhibit a significant impact (Figure 1A,B).

### 3.2. Hepatic Tissue Analysis via TUNEL Assay

**Figure 2:**
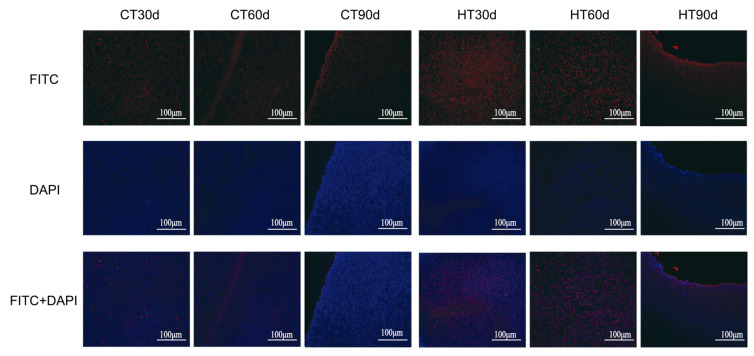
Fluorescent sections of TUNEL apoptosis in liver tissue of *C. fuscus* at 30, 60, and 90 days of chronic heat stress (*n* = 3). HT: high-temperature group; CT: control group. Note: The microscope magnification was 200×, and positive apoptotic cell nuclei are colored in red.

The hepatic tissues were subjected to TUNEL staining to assess cellular apoptosis following sustained high-temperature stress. The assay revealed an increased incidence of apoptotic cells in the liver tissues of *C. fuscus* at 30, 60, and 90 days of heat stress compared to the control group (Figure 2).

### 3.3. Effect of Chronic High Temperature on Biochemical Indexes in Serum and Liver of C. fuscus

**Figure 3:**
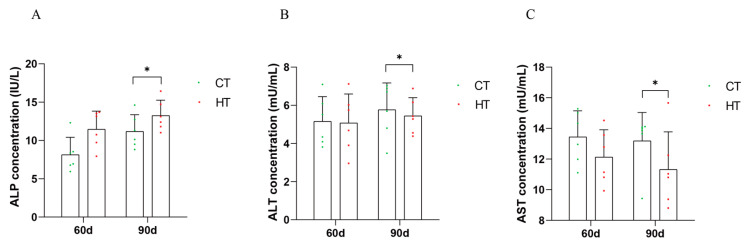
The levels of biochemical indexes in serum at different treatment stages were mean ± SD (*n* = 6). (**A**) alkaline phosphatase (ALP); (**B**) aspartate aminotransferase (AST); (**C**) alanine transaminase (ALT). HT: high-temperature group; CT: control group. * Indicated that there was a statistical difference between HT group and CT group at the same time point (*p* < 0.05).

The antioxidant enzyme concentration of serum immune index liver of *C. fuscus* during continuous high-temperature stress was analyzed. The results indicated that at day 90, the concentration of alkaline phosphatase (ALP) in the high-temperature group was significantly increased compared to the control group (Figure 3A). Conversely, the concentrations of alanine aminotransferase (ALT) and aspartate aminotransferase (AST) were significantly reduced (Figure 3B,C).

**Figure 4:**
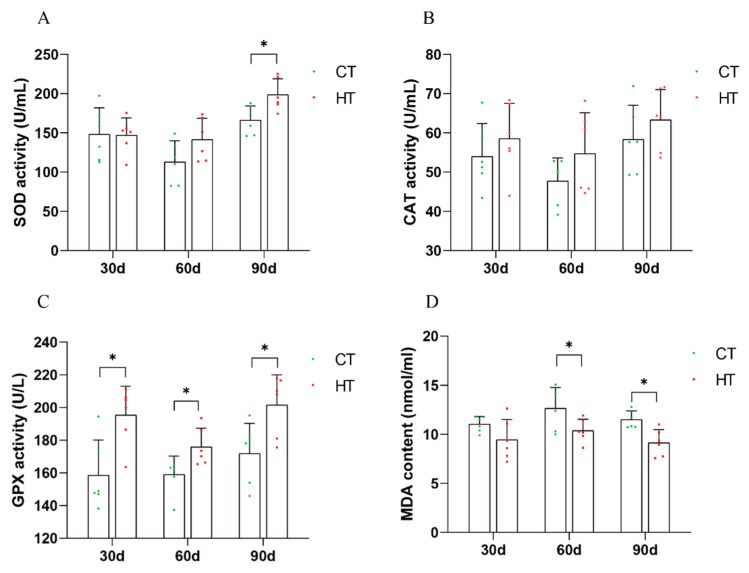
The levels of biochemical indexes in liver tissue at different treatment stages were mean ± SD (*n* = 6). (**A**) superoxide dismutase activity (SOD); (**B**) catalase activity (CAT); (**C**) glutathione peroxidase activity (GPx); (**D**) malondialdehyde content (MDA). HT: High temperature group; CT: control group. * Indicated that there was a statistical difference between HT group and CT group at the same time point (*p* < 0.05).

After a period of 90 days of thermal stress, the activity of superoxide dismutase (SOD) in the liver of *C. fuscus* was significantly higher than that observed in the control group (Figure 4A). In contrast, the activity levels of catalase (CAT) remained statistically unchanged at all three time points during the high-temperature treatment compared with the control group (Figure 4B). In comparison to the control, the activity of glutathione peroxidase (GPx) was significantly increased during the three periods of high temperature treatment (Figure 4C). Moreover, the concentration of malondialdehyde (MDA), an indicator of lipid peroxidation, was significantly reduced at the 60- and 90-day marks of the heat-stress period (Figure 4D).

### 3.4. Transcriptome Sequencing Analysis

#### 3.4.1. Sequencing of Reads in *C. fuscus* Liver

Twelve samples from the CT and HT groups were sequenced, resulting in a total of 77.48 Gb of high-quality clean reads. The HT and CT groups contributed 39.16 Gb and 37.28 Gb, respectively. The percentage of Q30 bases exceeded 92.6%, and the GC content ranged from 44.25% to 46.58%. These clean reads were compared to the genome sequence of *C. fuscus*, with a comparison rate of over 85.86% (Table S2). These findings demonstrate that the data obtained from this experiment are of high quality and can be relied upon for further analysis.

#### 3.4.2. Analysis of Differentially Expressed Genes (DEGs)

**Figure 5:**
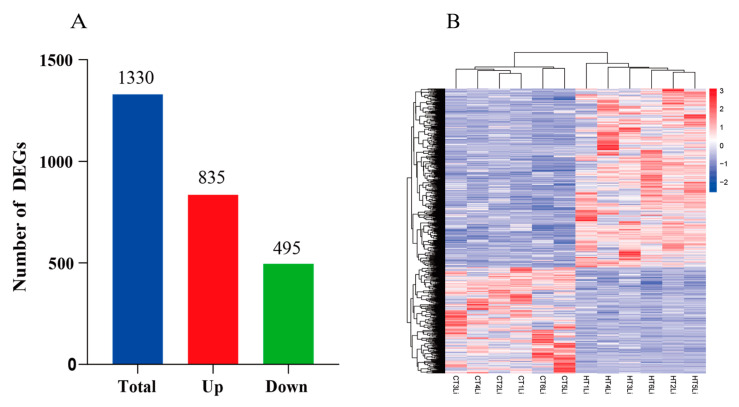
The number of DEGs was compared between CT group and HT group at 90 days of chronic heat stress. (**A**) Histogram of HT vs. CT differential genes. The vertical axis represents the number of genes, and red and blue represent upregulated and downregulated DEGs, respectively. (**B**) Hierarchical clustering analysis of DEGs. (**B**) Heatmap analysis of all DEGs hierarchical clustering in CT group and HT group. The log2 (FPKM + 1) value was normalized and transformed (scale number) and clustered. Red indicated high gene expression, and blue indicated low gene expression. The color is from red to blue, indicating that log2 (FPKM + 1) is from large to small. The liver tissue samples from the high-temperature-stress group were labeled as HT1Li, HT2Li, HT3Li, HT4Li, HT5Li, and HT6Li. The liver tissue samples from the control group were labeled as CT1Li, CT2Li, CT3Li, CT4Li, CT5Li, and CT6Li.

The changes in gene expression in liver tissue samples at 90 days in the high-temperature-stress group (HT) and control group (CT) were compared. A total of 1330 differentially expressed genes were identified, of which 835 genes were upregulated and 495 genes were downregulated (Figure 5A). The expression patterns of DEGs in the three treatment stages of CT group and HT group were determined via heat map (Figure 5B). Table 2 presents the top 20 genes ranked by fold change between the experimental and control groups.

#### 3.4.3. Functional Classification of DEGs

**Figure 6:**
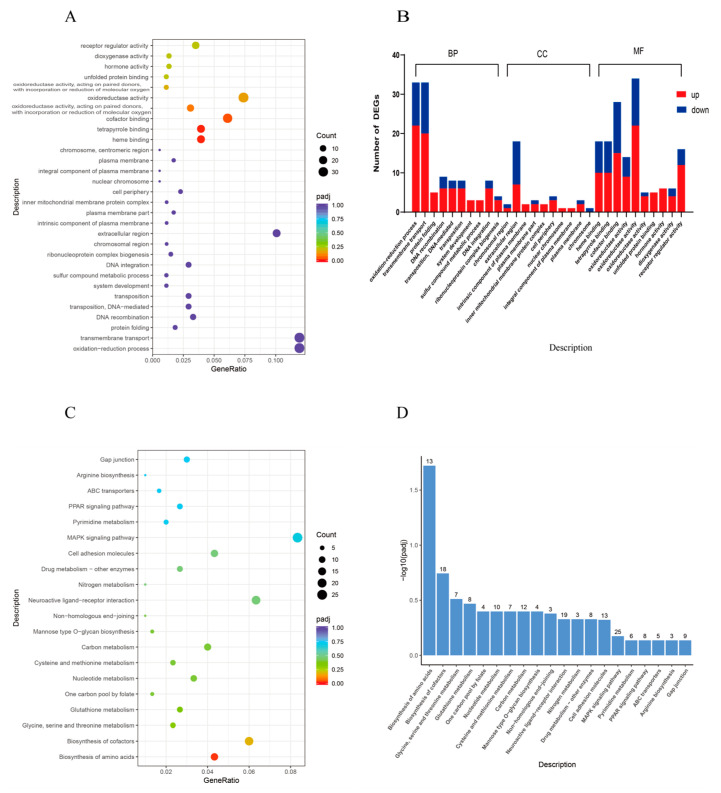
GO and KEGG enrichment analysis of differentially expressed genes in the liver tissue of *C. fuscus* under chronic heat stress. (**A**,**B**) Bubble maps and bar chart of the 10 most significant terms in terms of molecular function, cell composition, and biological processes of GO; (**C**,**D**) bubble maps and bar chart of top 20 important terms for KEGG enrichment.

To gain further insights into the biological significance of the DEGs, GO enrichment analysis was conducted. According to GO enrichment analysis, DEGs in the HT vs. CT group were significantly enriched into three functional categories, revealing 296, 74, and 227 enriched subcategories within the biological process (BP), cellular component (CC), and molecular function (MF) categories, respectively. The DEGs were significantly enriched in heme binding (GO:0020037), tetrapyrrole binding (GO:0046906), cofactor binding (GO:0048037), the number of enrichment in oxidation-reduction process (GO:0055114), transmembrane transport (GO:0055085), and oxidoreductase activity (GO:0016491) (Figure 6A,B).

Through KEGG analysis, the DEGs were enriched in pathways such as the biosynthesis of amino acids; biosynthesis of cofactors, glycine, serine and threonine metabolism, glutathione metabolism; and one carbon pool by folate and nucleotide metabolism. Among them, the amino acid biosynthesis pathway was significantly enriched, the number of enrichment in the MAPK signaling pathway is the highest (Figure 6C,D). The protein processing in endoplasmic reticulum pathway in the endoplasmic reticulum plays an important role in high-temperature stress. In this study, the genes enriched in this pathway are mainly related to heat shock proteins. The DEGs enriched in amino acid biosynthesis and protein processing in endoplasmic reticulum are listed in Table 3.

### 3.5. qRT-PCR Validation of Transcriptome Data

**Figure 7:**
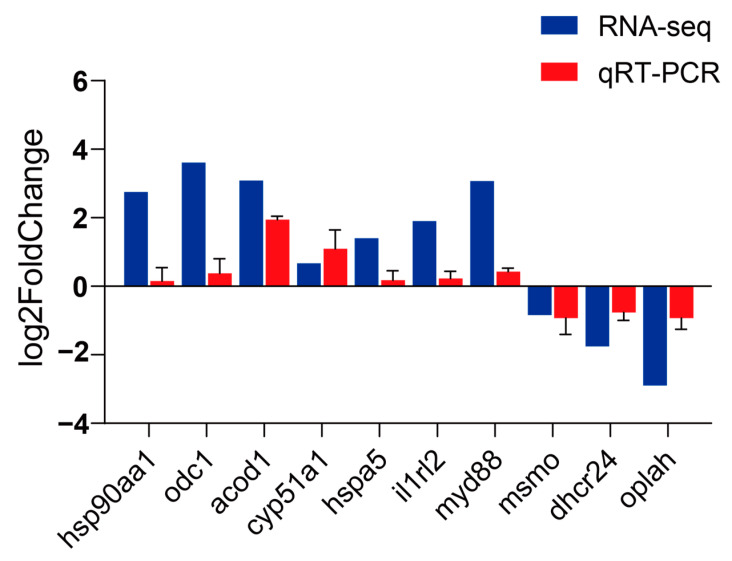
Comparison of expression levels of DEGs detected by qPCR and RNA-seq in *C. fuscus* liver.

Ten genes were randomly selected for qRT-PCR verification: *hsp90aa1*, *odc1*, *acod1*, *hspa5*, *cyp51a1*, *il1rl2*, *myd88, msmo*, *dhcr24*, and *oplah*. The expression patterns of all the selected genes were consistent in RNA-seq and qRT-PCR (Figure 7), indicating the accuracy and reliability of RNA-seq.

## 4. Discussion

Global warming presents a substantial global threat to life. Temperature, a critical non-biological factor, plays a significant role in influencing the well-being of animals [1]. Interestingly, animals with varying temperature tolerance demonstrate remarkable adaptability to a wide range of temperatures [41]. Changes in water temperature have profound effects on fish physiology and metabolism [42]. Further research is needed to investigate the molecular biological mechanism underlying their response to high temperatures. Consequently, studying the response mechanism of fish to rising ambient temperatures has emerged as a prominent area of interest.

The growth and metabolism levels of aquatic organisms are highly influenced by environmental temperature and exhibit stronger stress responses [43]. The growth rate tends to increase with rising ambient temperature and reaches its peak under optimal thermal conditions. However, if the temperature deviates from this optimal range, the growth rate is hindered [44]. The previous studies have indicated that the suitable survival water temperature for *C. fuscus* ranges from 10 to 32 °C, with the optimal growth temperature being between 25 and 30 °C [33]. The findings of this study revealed that prolonged exposure to high temperatures (34 °C) significantly slowed down the growth rate of *C. fuscus* compared to the control group. This aligns with the existing evidence suggesting that fish reduce their food intake during periods of high temperature, resulting in lower energy allocation for growth and subsequently lower growth rates [45]. Similar observations have been made in other fish species. Li et al. [46] reported a significant reduction in the growth performance of medaka larvae reared at temperatures as high as 32 °C. In this study, it was found by observing the liver tissue of *C. fuscus* via TUNEL staining that chronic high temperature could not only inhibit the growth of catfish but also lead to apoptosis of tissue cells. The results are consistent with Wang et al.’s research on pufferfish (*Takifugu obscurus*) [21,47].

Elevated temperatures have been shown to affect enzymatic activities that indicate the health of fish [33]. When tissues or organs are damaged, specific enzymes are released into the bloodstream, acting as biomarkers for the physiological condition [48,49]. Aspartate aminotransferase (AST) and alanine aminotransferase (ALT) are well-known for their role in indicating liver function in fish [33,50]. In our study, we observed a decrease in AST and ALT concentration in *C. fuscus* in response to thermal stress, suggesting a potential suppression of hepatic metabolism or cellular damage. On the other hand, alkaline phosphatase (ALP), an enzyme involved in calcium and phosphorus homeostasis, as well as immune and metabolic functions, showed increased activity under high-temperature stress. This enzyme has detoxification, defense, and digestion functions and is a sign of fish health [51]. In fish, ALP is mainly secreted by organs such as the liver, intestine, and kidneys. This increase may indicate a compensatory mechanism or an adaptive response to maintain physiological balance. Our findings support the previous research conducted on other species such as *Ctenopharyngodon idellus*, *Horabagrus brachysoma*, and *Gymnocypris chilianensis* [42,52,53], which also exhibited similar enzymatic responses to elevated temperatures.

High temperatures can lead to oxidative stress, which can ultimately result in apoptosis [54]. Antioxidant enzymes play a crucial role in defending against oxidative stress and are essential for innate immunity through redox signaling [55]. When stress increases mitochondrial respiration, it intensifies the generation of reactive oxygen species (ROS), which triggers the upregulation of SOD, CAT, and GPx at both the translational and transcriptional levels to counteract oxidative damage [56,57]. However, CAT activity may decrease due to competitive inhibition from GPx, as both enzymes counteract H_2_O_2_-induced oxidative harm [58]. Studies have shown that high-temperature stress in fish can increase ROS levels, leading to protein alteration, lipid peroxidation, and DNA damage [59,60]. The enzymatic responses to heat stress vary [61]. In this study, the activities of antioxidant enzymes (SOD, CAT, and GPx) in the liver of the high-temperature group were consistently increased during continuous high-temperature stress, compared to the control group. This finding is consistent with the previous research [62,63], suggesting that *C. fuscus* may enhance its ability to eliminate ROS by increasing the activity of these enzymes under heat stress. Malondialdehyde (MDA) is a byproduct of the reaction between ROS and unsaturated fatty acids in the cell membrane [64]. The change in MDA content in tissues indirectly reflects the level of oxidative stress and damage caused by excessive ROS to the cell membrane [65]. Excessive ROS can react with membrane lipids to produce MDA [66]. Wang et al. [22] investigated the oxidative stress in pike-perch liver and found that MDA content decreased with continuous heat stress. Similarly, in our study, the liver MDA content showed a decreasing trend at each period when compared to the control group, indicating a reduction in lipid peroxidation and cell damage [67]. Furthermore, the TUNEL fluorescence section observation in this study confirmed that the apoptosis signal induced by high-temperature stress for 90 days was weakened (Figure 2).

Transcriptomic analyses have played a crucial role in understanding the impact of thermal stress on fish species, such as *Oncorhynchus mykiss* [68] and *Salmo salar* [69]. The liver, which is an important organ in maintaining metabolic balance and regulating amino acid and carbohydrate metabolism, is essential for adapting to heat stress [23]. Various key pathways, including metabolism, proteostasis, and immune function, are involved in thermal tolerance [27]. This investigation supports the previous findings that show that elevated temperatures lead to significant changes in genes related to amino acid synthesis and endoplasmic reticulum protein processing. Similar transcriptomic responses have been observed in different species facing similar thermal challenges, such as *Oncorhynchus mykiss* [70], *Cynoglossus semilaevis* [71], and *Schizothorax richardsonii* [72].

The biosynthesis of amino acids is closely connected to the central metabolism of carbon, nitrogen, and sulfur [73]. This study found a significant enrichment of amino acid biosynthesis pathways, including in genes such as *mat1a*, *mat2a*, *BCAT2*, and *ENO3*. These genes have been associated with iron death. Ferroptosis, which is known to play an important role in inflammation [74], was also observed. The previous studies have shown that reduced activity of liver-specific MAT is linked to the development of liver disease and increased susceptibility to liver damage. This is supported by studies demonstrating increased liver vulnerability and cell proliferation when mat1a is knocked out in mice [75]. In the present study, it was found that mat1a and mat2a were significantly upregulated in the liver under prolonged high-temperature conditions. This suggests that the expression of mat1a and mat2a is induced to protect liver tissues from heat-stress injury. Additionally, the study found that branched chain aminotransferase 2 (BCAT2), which is involved in sulfur-containing amino acid metabolism, plays a role in regulating intracellular glutamate levels and counteracting the inhibition of the Xc-system. This provides protection to hepatocellular and pancreatic carcinoma cells against ferroptosis both in vitro and in vivo [76]. Wang et al. reported that downregulation of BCAT2 transcripts reduces glutamate levels, promoting ferroptosis in hepatocellular carcinoma cells [77]. This study shows that increased BCAT2 expression can slow ferroptosis in hepatocytes. Enolase (ENO3), a metalloenzyme essential for glycolysis, is abnormally expressed in various cancers, and its overexpression has been shown to mitigate ferroptosis [78]. In the context of high-temperature stress, *C. fuscus* appears to regulate the expression of genes associated with ferroptosis, including *mat1a*, *mat2a*, *BCAT2*, and *ENO3*, in order to minimize liver damage and protect against iron death. The upregulation of iron-death-related genes was observed in the liver tissues of high-temperature-treated *C. fuscus*, indicating that *C. fuscus* has the ability to regulate its own metabolism to mitigate liver tissue damage and protect against iron death.

Apoptosis, a critical process of programmed cell death, is essential for development and immune function in organisms. The previous studies have shown that heat stress can increase levels of reactive oxygen species (ROS) in aquatic life, and excessive ROS can trigger apoptosis [46]. In the present study, it was found that sustained high-temperature-induced oxidative stress led to tissue damage and, hence, apoptosis. The results are consistent with Cheng et al. [47]. The protein GADD45α, which belongs to the growth arrest and DNA-damage-inducible protein family, plays a crucial role in regulating the cell cycle, maintaining genomic stability, and inducing apoptosis [79]. In this study, the expression of GADD45α was significantly upregulated, indicating that apoptosis occurred in the liver tissue of *C. fuscus* under continuous high-temperature stress, which aligns with the TUNEL results mentioned above. Taken together, these observations indicate that high-temperature stress leads to apoptosis and liver injury in *C. fuscus*.

In our study, we observed that exposure to heat stress at 34 °C significantly alters the protein processing within the endoplasmic reticulum (ER) of *C. fuscus*. This alteration is supported by the upregulation of heat shock proteins (HSPs), specifically Hsp40 (*dnaja1*), Hsp70 (*hspa8*, *hyou1*), and Hsp90 (*hsp90αα1*), which is consistent with previous findings [22,70,80,81]. These HSPs play a crucial role in mitigating the effects of stress by facilitating the refolding of denatured proteins and eliminating irreparable ones [82]. The Hsp40 family, through its co-chaperone activity, enhances the ATPase function of Hsp70, thereby promoting effective protein folding [83]. Hsp70 and Hsp90 have been extensively studied for their stress-responsive increase in expression and their pivotal role in cellular protection against various stressors [84,85]. Additionally, HSPs contribute to cell survival, potentially by preventing apoptosis [86]. The induction of these HSPs indicates that *C. fuscus* employs an adaptive mechanism to maintain proteostasis and confer resistance against heat-induced damage.

## 5. Conclusions

Chronic heat stress has been found to inhibit the growth of *C. fuscus* and cause damage to its liver, leading to oxidative stress and resulting in DNA damage. This chronic heat-stress response after 90d of high temperature (34 °C) treatment involves important pathways such as the biosynthesis of amino acids and protein processing in endoplasmic reticulum. In order to maintain cellular homeostasis, the organism upregulates the activities of antioxidant enzymes (SOD, CAT, GPx) and the expression of heat shock protein (HSP) genes as a protective response. The further investigation of these adaptive pathways can enhance our understanding of the mechanisms underlying thermal tolerance in *C. fuscus* under prolonged heat-stress conditions.

## Figures and Tables

**Table 1:** Quantitative real time PCR (qRT-PCR) primer.

Gene Name	Primer Sequences (5′-3′)
*Hsp90aa1*	F: GGACCATCGCCAAAT
	R: GGCAGAGTAGAAACCCAC
*Odc1*	F: GGATCATTTATGCCAACC
	R: TCCGAGCAACCTTCATT
*Acod1*	F: CGACCTTACGACGTGAACAT
	R: TTTAGCCAGACCCAACACAC
*Cyp51a1*	F: ACGACGATGAGATTTCCG
	R: CTTGCTTCTGTTCGCTGTA
*Hspa5*	F: ACGGGCAACAAGAACAAGAT
	R: GGTTCTTGAGTGAGTAGGCG
*Il1rl2*	F: GGGTGTCTAAGGGTTGG
	R: GCACTTCCTCTGTGGGTT
*Myd88*	F: GAGAGAATCCCACGCAGAAG
	R: TTATGCTTCGATCCTGGTGC
*Msmo*	F: AGGCTTCGTGTTCCAGTT
	R: CCAGTCGTAAGGGATGCTA
*Dhcr24*	F: AGTGGTGTTTGATGCCTAC
	R: TTCCTCCCTCCTTCTTC
*Oplah*	F: TTCACCGCACGCTACAT
	R: GGCCAAAGTCCCGATT
*β-actin*	F: AGGCTGGATTCGCTGGAGATGAT
	R: TGGTGACAATACCGTGCTCAATGG

**Table 2:** Top 20 DEGs in HT vs. CT group of *C. fuscus* under high-temperature stress.

Gene ID	Gene Name	Gene Annotation	log2 (FC)
gene-Cfus20961	*gprin3*	G protein-regulated inducer of neurite outgrowth 3	8.3369
gene-Cfus12438	*chs2*	chitin synthase2	8.1574
gene-Cfus21522	*pprc1*	Peroxisome proliferator-activated receptor gamma coactivator-related protein 1	8.0864
gene-Cfus01065	*robo2*	roundabout homolog 2-like	7.7606
gene-Cfus18848	*entpd2*	ectonucleoside triphosphate diphosphohydrolase 2	7.2640
gene-Cfus18283	*ptx*	pentraxin fusion protein-like	6.8802
gene-Cfus01755	*porcn*	protein-serine O-palmitoleoyltransferase porcupine-like	6.8777
gene-Cfus03155	*tuba1c*	tubulin alpha-1C chain	6.7665
gene-Cfus03755	*arpp21*	cAMP regulated phosphoprotein 21	6.5925
gene-Cfus06190	*trim25*	E3 ubiquitin/ISG15 ligase TRIM25-like	6.4865
gene-Cfus08092	*LOC106488566*	RNA-directed DNA polymerase from mobile element jockey-like	−6.7983
gene-Cfus13395	*cacna1h*	voltage-dependent T-type calcium channel subunit alpha-1H-like	−6.6817
gene-Cfus06402	*nceh1*	neutral cholesterol ester hydrolase 1-like	−6.4654
gene-Cfus20062	*cps1*	carbamoyl-phosphate synthase 1	−6.2394
gene-Cfus02255	*ankrd13d*	ankyrin repeat domain 13D	−5.9840
gene-Cfus15168	*chrna2*	neuronal acetylcholine receptor subunit alpha-2-like	−5.4795
gene-Cfus19983	*lrrc58*	leucine rich repeat containing 58	−5.4091
gene-Cfus13163	*vmo1*	vitelline membrane outer layer protein 1 homolog	−5.2657
gene-Cfus16454	*mast2*	microtubule associated serine/threonine kinase 2	−5.0676
gene-Cfus17407	*sbk1*	serine/threonine-protein kinase SBK1-like	−4.8348

**Table 3:** DEGs enriched for biosynthesis of amino acids and protein processing in endoplasmic reticulum. Up represents upregulated genes, and down represents downregulated genes.

Pathway/ Gene Name	Gene Annotation	log2 (FC)	Up/ Down
Biosynthesis of amino acids			
*mat2a*	Methionine adenosyltransferase 2A	3.0678	Up
*mat1a*	S-adenosylmethionine synthase isoform type-1	2.2197	Up
*bcat2*	Branched-chain-amino-acid aminotransferase, mitochondrial	2.4590	Up
*eno3*	Enolase 3	2.1771	Up
*shmt1*	Serine hydroxymethyltransferase, cytosolic-like	2.0500	Up
*srr*	L-threonine ammonia-lyase-like	2.8882	Up
*arg2*	Arginase-2, mitochondrial	2.2139	Up
*psat1*	Phosphoserine aminotransferase 1	−2.1117	Down
*phgdh*	Phosphoglycerate dehydrogenase	−2.1841	Down
*tkt*	Transketolase	−2.6636	Down
*cps1*	Carbamoyl-phosphate synthase 1	−6.2394	Down
Protein processing in endoplasmic reticulum			
*hsp90a.1*	Heat shock protein HSP 90-alpha	2.7554	Up
*hspa8*	Heat shock cognate 71 kDa protein	2.0996	Up
*hyou1*	Hypoxia upregulated 1	2.2399	Up
*ube2g1*	Ubiquitin-conjugating enzyme E2 G1-like	2.1523	Up
*cryaa*	Crystallin alpha A	2.7842	Up
*ern2*	Endoplasmic reticulum to nucleus signaling 2	3.5579	Up
*dnajai*	DnaJ homolog subfamily A member 1-like	2.8502	Up

## Data Availability

The raw data of Illumina transcriptome have been submitted in the SRA under accession number PRJNA988888.

## Supplementary Materials

The following supporting information can be downloaded at: https://www.mdpi.com/article/10.3390/ani14071006/s1, Table S1: Quantitative real time PCR (qRT-PCR) primer sequences data. Table S2: Sequencing statistics of the liver transcriptome of *Clarias fuscus.*
